# Oncogenic Signalling through Mechanistic Target of Rapamycin (mTOR): A Driver of Metabolic Transformation and Cancer Progression

**DOI:** 10.3390/cancers10010005

**Published:** 2018-01-03

**Authors:** Ellie Rad, James T. Murray, Andrew R. Tee

**Affiliations:** 1 Division of Cancer and Genetics, Cardiff University, Heath Park, Cardiff CF14 4XN, UK; rade@tcd.ie; 2 School of Biochemistry & Immunology, Trinity Biomedical Sciences Institute, Trinity College Dublin, Dublin 2, Ireland; james.murray@tcd.ie

**Keywords:** mTOR, cancer, cell growth, S6K1, 4E-BP1, eIF4E, HIF, STAT3, SGK1

## Abstract

Throughout the years, research into signalling pathways involved in cancer progression has led to many discoveries of which mechanistic target of rapamycin (mTOR) is a key player. mTOR is a master regulator of cell growth control. mTOR is historically known to promote cell growth by enhancing the efficiency of protein translation. Research in the last decade has revealed that mTOR’s role in promoting cell growth is much more multifaceted. While mTOR is necessary for normal human physiology, cancer cells take advantage of mTOR signalling to drive their neoplastic growth and progression. Oncogenic signal transduction through mTOR is a common occurrence in cancer, leading to metabolic transformation, enhanced proliferative drive and increased metastatic potential through neovascularisation. This review focuses on the downstream mTOR-regulated processes that are implicated in the “hallmarks” of cancer with focus on mTOR’s involvement in proliferative signalling, metabolic reprogramming, angiogenesis and metastasis.

## 1. Introduction

Cancer is a complex disease and is known to be one of the leading causes of mortality in the modern world. mTOR is referred to as a master regulator of cell growth control and is often activated in cancer. mTOR is estimated to be aberrantly activated in over 70% of cancers [1]. mTOR functions as a serine/threonine protein kinase that was initially discovered as a drug target of rapamycin. mTOR is classically known to drive cell growth through the regulation of protein translation. However, we are now beginning to appreciate that mTOR exerts its control on cell growth in a much more multifaceted manner [2]. mTOR is centrally involved in building up cellular bio-mass, which is rate-limiting for hyper-proliferative cancer cells. A cancer cell’s capacity to grow and proliferate is often restricted by the limited supply of pre-cursor molecules necessary to generate proteins, lipids and nucleotides (reviewed in [3]). mTOR helps generate proteins, lipids, and nucleotides through the promotion of anabolic processes, while turning off catabolic processes such as autophagy [4]. When mTOR is active, the capacity of the cell to manufacture *de novo* protein is greatly enhanced through the generation of ribosomes (via ribosomal biogenesis) and increased rates of protein translation (reviewed in [5]). More recently, mTOR was shown to be involved in lipid generation [6] as well as the biosynthesis of nucleotide precursors [7,8], which are required for a growing cell to expand their membrane and to generate nucleotides for ribonucleic acid (RNA) transcripts and DNA. mTOR is also involved in metabolic transformation, neovascularisation and metastasis. Given the broad range of cancerous attributes that are promoted by mTOR, it is not surprising that cancer cells hijack the mTOR pathway as a mechanism to drive their progression. For example, in cancer patients, mTORC1 activation often correlates with metastasis, poor patient survival and resistance to anticancer agents [9]. This review will outline the current understanding of how mTOR signaling contributes to oncogenesis and disease progression.

## 2. mTOR and Cancer

### 2.1. mTOR Complexes and the Upstream Signalling Pathways

In mammalian cells, mTOR functions as two distinct protein kinase complexes, mTOR complex 1 (mTORC1) and mTORC2, which can be distinguished by their differences in rapamycin sensitivity, core binding proteins and downstream substrates [10]. mTORC1 was first identified by the association of the catalytic mTOR subunit with the scaffolding protein rapamycin-associated protein of TOR (Raptor) and mammalian lethal with SEC13 protein 8 (mLST8). Raptor defines the substrate specificity of mTORC1 to recruit substrates and presents them to the kinase active site of mTOR for their efficient phosphorylation (reviewed in [11]). mTORC1 also associates with two negative regulators; proline-rich substrate of 40 kDa (PRAS40) [12] and Dishevelled, EGL-10 and Pleckstrin (DEP) domain-containing mTOR-interacting protein (DEPTOR) [13]. Overlapping binding components that are also integral to mTORC2 include LST8 and DEPTOR, while Raptor-independent companion of mTOR (Rictor), Stress-activated map kinase Interacting Protein 1 (SIN1) and protein observed with rictor-1 (PROTOR-1) are core binding subunits that are exclusive to mTORC2 [14,15]. Rictor is essential for the assembly and stabilisation of mTORC2 as well as the substrate specificity of this complex, while SIN1 acts as a negative regulator of mTORC2 [16]. Localisation of these two mTOR complexes are also distinct. mTORC1 associates with endosomal and lysosomal membranes, whereas mTORC2 interacts with the plasma membrane and in some cases to ribosome-associated membranes, such as the rough endoplasmic reticulum (ER). mTORC1 is regulated by both nutrient, energy and growth signalling inputs, while mTORC2 is activated via growth signals. One key difference between the mTOR complexes is their differential sensitivity to the allosteric inhibitor, rapamycin. mTORC1 is sensitive to rapamycin while mTORC2 shows initial resistance to rapamycin over short periods of treatment [17]. Rapamycin treatment over longer time periods can inhibit mTORC2 signalling by binding to “free mTOR”, preventing mTOR′s association with Rictor to block mTORC2 complex assembly. Prolonged (24 h) rapamycin treatment results in saturation of the newly synthesised mTOR with rapamycin binding, causing a suppression of mTORC2 and AKT serine/threonine kinase (AKT) signalling [17]. This effect appears to be variable between cell-types with some being more sensitive to inhibition of mTORC2 assembly with rapamycin than others. The variability of rapamycin sensitivity could also be due in part by signalling cross-talk between mTOR complexes. It was found that the p70 ribosomal protein S6 kinase 1 (S6K1), a downstream substrate of mTORC1, phosphorylates Rictor to inhibit mTORC2 [18]. Therefore, inhibition of mTORC1 and S6K1 could lead to enhanced activity of mTORC2 during short treatments with rapamycin.

**Figure 1:**
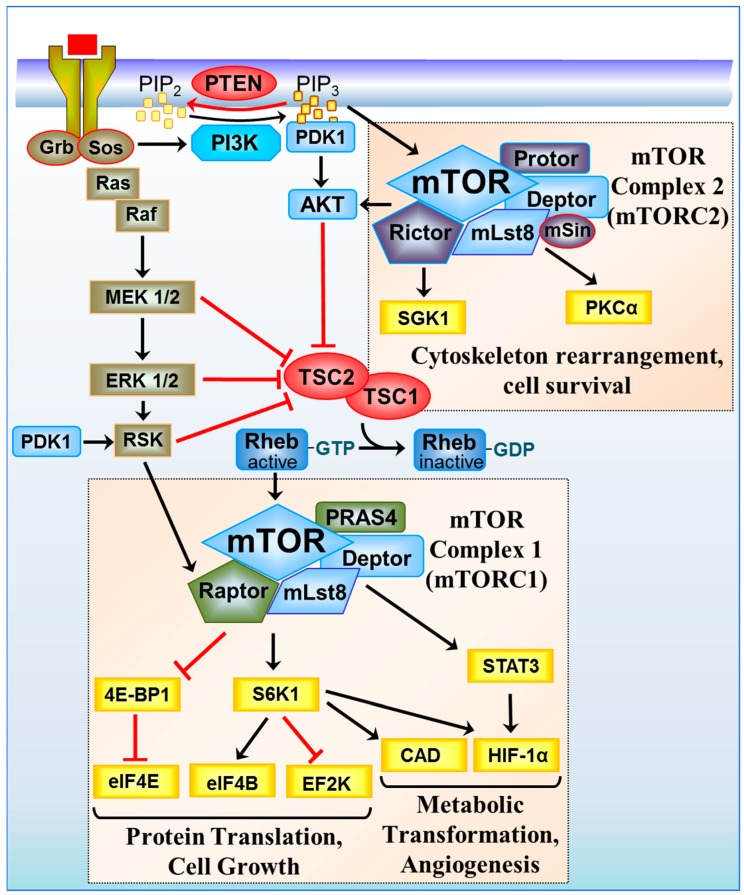
Signal transduction through the mTOR complexes. Growth signals from tyrosine receptor kinases are relayed through the Phosphoinositide 3-kinase (PI3K)/phosphoinositide-dependent kinase 1 (PDK1)/AKT and Ras (Rat sarcoma) signalling pathway to inhibit the tumour suppressor TSC1/TSC2. TSC1/TSC2 acts as a Ras homolog enriched in brain GTPase activating protein (RhebGAP), converting active Rheb-GTP to an inactive GDP-bound state. When TSC1/TSC2 is turned off, Rheb is GTP-bound, and mTORC1 is activated to promote cell growth. mTORC1 regulates protein translation through Eukaryotic translation initiation factor 4E-binding protein 1/Eukaryotic translation initiation factor 4E (4E-BP1/eIF4E) and S6K1 and eukaryotic translation initiation factor 4B/eukaryotic elongation factor 2 kinase (eIF4B/EF2K), inducing metabolic transformation through the regulation of signal transducer and activator of transcription 3/hypoxia inducible factor-1α (STAT3/HIF-1α) and carbamoyl-phosphate synthetase 2, aspartate transcarbamylase, and dihydroorotase (CAD). HIF-1α protein synthesis is also upregulated in an eIF4F and S6K1-dependent manner. mTORC2 regulates the cytoskeleton and cell survival through serum and glucocorticoid-regulated kinase 1 (SGK1) and protein kinase Cα (PKCα).

Aberrant mTOR signalling in cancer is commonly caused by either loss of function mutations of upstream tumour suppressor proteins or activating mutations within oncogenes that feed into the mTOR pathway (depicted in Figure 1). Research on inherited hamartoma syndromes has helped delineate the mTOR signalling pathway, where constitutive mTOR activation plays a pivotal role in their disease pathology and tumour predisposition. Loss-of-function mutations to Tuberous Sclerosis Complex 1 (TSC1) and TSC2 are responsible for the hamartoma condition, TSC [19]. TSC1 and TSC2 are also mutated in bladder cancer, clear cell renal carcinoma and well-differentiated pancreatic neuroendocrine tumours, but at a low frequency [20,21,22]. TSC1 and TSC2 negatively regulate mTORC1 by acting as a GTPase activating protein (GAP) towards the small G-protein, Ras homolog enriched in brain (Rheb) [23,24]. TSC1/TSC2 inhibits mTORC1 indirectly by reverting Rheb to an inactive GDP-bound state. When TSC1/TSC2 is negatively regulated via growth signalling inputs or is functionally inactivated through mutation, Rheb becomes GTP-bound. Rheb switches to an activate state when GTP-bound, causing Rheb to bind to and activate mTORC1. While mutation to TSC1/TSC2 and mTOR are rare occurrences in cancer, mutation to components higher in the signalling pathway are much more common [25,26]. For instance, the tumour suppressor PTEN (phosphatase and tensin homolog deleted on chromosome 10) is the second most frequently mutated gene in human cancer, after TP53 [27].

Within the phosphoinositide 3-kinase (PI3K)/PTEN/AKT pathway, PTEN directly opposes the activity of PI3K through dephosphorylating phosphatidylinositol-3,4,5-triphosphate (PtdIns3,4,5P3) that drives downstream PI3K signalling events that feed onto both mTOR complexes (see Figure 1). The Ras proto-oncogene, GTPase (RAS)/RAF/ mitogen-activated protein kinase (MAPK) pathway is also commonly activated in cancer. RAF is regulated by three closely related RAS small G-protein family members, HRAS, KRAS and NRAS. In sporadic cancer, activating KRAS mutations are more frequent at 21.6%, when compared to either NRAS (8%) or HRAS (3.3%) [28]. Activated RAS binds to RAF, its downstream effector, causing re-localisation of RAF to the plasma membrane and signal transduction through the MAPK signalling cascade that includes activation of mitogen-activated protein kinase kinase (MEK), extracellular signal-regulated kinases 1 and 2 (ERK1/2) and ribosomal protein S6 kinase (RSK) (see Figure 1). Signal transduction through either one of these parallel pathways, PI3K/PTEN/AKT or RAS/RAF/MAPK/ERK/RSK, has the capacity to inactivate TSC1/TSC2 via phosphorylation of TSC2 by AKT [29], ERK [30] and RSK [31], which then results in the conversion of Rheb to a GTP-bound form and activation of mTORC1. RSK has also been shown to directly phosphorylate Raptor to further enhance the activity of mTORC1 [32]. Gene amplification of growth factor tyrosine kinase receptors that are upstream of both PI3K and RAS are also common occurrences in cancer that also leads to aberrant signal transduction through both mTOR complexes. Much more is known about mTORC1, which will be discussed first below.

### 2.2. mTORC1 Coordinates Cell Growth Control

mTORC1 is classically known to regulate protein translation via several translation factors that include eukaryotic initiation factor 4E-binding protein 1 (4E-BP1) and S6K1. 4E-BP1 and S6K1 are recognised by Raptor through an mTORC1 signalling (TOS) motif (a motif that follows the general composition F-E/D-M-D-I/L) and is necessary for Raptor interaction with substrates and subsequent phosphorylation by mTORC1 [33] 4E-BPs act as repressors of protein translation that when unphosphorylated will bind to and inhibit eukaryotic initiation factor (eIF) 4E at the m7GpppN cap moiety on the 5′-end of messenger RNAs (mRNAs) [34]. mTORC1-mediated phosphorylation of 4E-BP1 on four Ser/Thr residues causes its dissociation from eIF4E. 4E-BP1 dissociation allows eIF4E to sequentially associate with eIF4G, a scaffold protein that recruits an array of other translation initiation factors to form the eIF4F complex to promote translation initiation. eIF4A is an integral component of the eIF4F complex that functions as an RNA helicase to unwind the secondary structure within the 5′-untranslated region (UTR) of the mRNA to allow the ribosome to efficiently scan along the 5′-UTR from the 5′-cap structure to the AUG start codon (reviewed in [35]). Some mRNAs are more dependent on eIF4F than others to direct ribosomes to the start codon, where the length and the degree of secondary structure within the 5′-UTR contributes to this dependency [36]. Assembly of eIF4F is a rate-limiting step of translation initiation. In some cancers, eIF4E is over-expressed to enhance assembly of eIF4F, leading to transformation (reviewed in [37]). Expression of eIF4E is increased by three to 10-fold in head and neck, bladder, colon, breast, prostate, lung and blood cancers (reviewed in [37]). High expression levels of eIF4E increases the translation efficiency of a set of target mRNAs involved in cancer progression that are more dependent on eIF4F, which includes: (i) MYC proto-oncogene (MYC) and cyclin D1 (CCND1), both involved in proliferative drive, (ii) vascular endothelial growth factor A (VEGFA) that encourages angiogenesis, (iii) MCL1 (MCL1, BCL2 Family Apoptosis Regulator) and Survivin that are linked to cancer cell survival, (iv) snail family zinc finger 1 (SNAI1) involved in epithelial-to-mesenchymal transition (EMT), and (v) matrix metallopeptidase 3 (MMP3) that enhances metastasis (reviewed in [37]).

mTORC1 further promotes protein synthesis by phosphorylation and activation of S6K1. S6K1 was first identified as the kinase responsible for the phosphorylation of the 40S ribosomal protein S6 (rpS6) on Ser235/Ser236 and is rapamycin sensitive [38]. While the role that rpS6 has in the regulation of ribosomes is still unknown, rpS6 phosphorylation is still commonly used as a functional readout of S6K1 activity. Another target of S6K1 is eIF4B, which is a component of the eIF4F complex. eIF4B is phosphorylated on Ser422 by S6K1, increasing its association with eIF3 and enhances translation initiation through increasing the activity of eIF4A to unwind the mRNA secondary structure within the 5′-UTR (untranslated region) [39]. S6K1 also phosphorylates and inactivates eukaryotic elongation factor 2 kinase (eEF2K) [40]. As eEF2K is a negative regulator of the elongation phase of protein synthesis, S6K1 indirectly promotes translocation of the ribosome along the mRNA. S6K1 is also known to promote ribosomal biogenesis where over 75% of ribosomal biogenesis factors are controlled by S6K1 [41]. 

A major hallmark of cancer is proliferative drive, which usually involves the loss of cell cycle control and an accumulation of DNA damage, as cells are unable to arrest at cell cycle check-points. mTORC1 regulates the transition between G_1_-S of the cell cycle as cells finish their first growth phase and DNA synthesis is initiated. Showing the involvement of mTOR, over-expression of hyperactive mutants of mTOR speeds up G_1_-phase progression into S-phase, while treatment with rapamycin stalls G_1_-S progression [42]. Progression through the cell cycle is regulated by the build-up and breakdown of cyclins. CCND1 forms an active cyclin complex with cyclin-dependent kinase 4 (Cdk4) to stimulate cyclin E (CCNE)/CdK2 complex activation by altering the binding activity of the inhibitory cyclin-dependent kinase inhibitor 1B (CDKN1B, also known as p27Kip1) [43]. These active cyclin complexes phosphorylate the tumour suppressor protein, Retinoblastoma (Rb) on Ser795 [44], which leads to the activation of the transcription factor, E2F, and entry into S-phase. As stated before, the protein translation of CCND1 is enhanced with eIF4E over-expression and shows a dependency of eIF4F to promote the translation of CCND1 [37]. However, mTORC1 also further regulates the translation of CCND1 mRNA through S6K1. It was found that small interfering RNA (siRNA) knockdown of S6K1 caused a 20–30% reduction in CCND1 protein expression that was rescued when an active mutant of S6K1 was over-expressed [45]. Rapamycin treatment was observed to reduce the association of CCND1 mRNA with polysomes, revealing that mTORC1 enhances the recruitment of ribosomes to the CCND1 mRNA [45]. Cancer cells can markedly amplify the protein levels of CCND1 by either over-expressing eIF4E or aberrantly activating mTORC1 (or both) and consequently are able to accelerate through the G1-S phase of the cell cycle.

mTORC1 also regulates the translation of mRNAs containing 5′ terminal oligopyrimidine (5′-TOP) tracts. The 5′-TOP functions as a translational cis-regulatory element consisting of pyrimidine nucleotides, and gives the mRNA sensitivity to rapamycin via a mechanism that is currently unknown. Such 5′-TOP elements are found in mRNA that encode ribosomal proteins and translation initiation factors involved in ribosomal biogenesis [46,47]. High resolution ribosomal profiling revealed that 144 mRNAs were acutely sensitive to mTORC1 inhibitors [48]. Of the 144 mTORC1-sensitive target genes uncovered, 68% possessed a 5′-TOP and 63% also possessed a newly discovered pyrimidine-rich translational element (PRTE). It was found that this PRTE conferred their sensitivity to mTORC1 inhibitors via 4E-BP1. Further work is required to understand how mTORC1 and 4E-BP1 regulate these mRNAs containing either 5′-TOP or/and PRTEs. Of note, genes associated with pro-invasion and metastasis were found within the list of mTORC1-sensitive mRNAs and included YB1 (Y-box binding protein 1), vimentin, MTA1 (metastasis-associated 1), and CD44 [48]. 

Many of the mTORC1-sensitive target genes uncovered within the Hsieh et al. study are ribosomal proteins [48]. The *de novo* synthesis of ribosomes is vastly enhanced during cell growth in hyper proliferative cells and requires a significant amount of energy, amino acids, and nucleotides. Given the importance of controlling the number of ribosomes in a growing cell, cells have developed several mechanisms by how mTOR regulates their assembly. Gene expression of these ribosomal proteins is also upregulated by a transcriptional mechanism through Split Finger Protein 1 (SFP1), which is rapamycin-sensitive [49]. mTORC1 also transcriptionally regulates ribosomal biogenesis via three nuclear RNA polymerases: RNA polymerase I (Pol I) to transcribe ribosomal RNA (rRNA), Pol II to produce ribosomal proteins, and Pol III for the synthesis of transfer RNA (tRNA) and 5S RNA. mTORC1 positively regulates the transcription of ribosomal RNA via Pol I activation. mTORC1 does this through several regulatory factors of Pol I, Transcription Initiation Factor 1A (TIF1A) and TIF1B. mTORC1 phosphorylates TIF1A and is required for its nucleolus localisation and activation of Pol I [50]. TIF1B is indirectly regulated by mTORC1 via S6K1, where S6K1 phosphorylates Upstream Binding Factor (UBF), which is required for UBF interaction with TIF1B and Pol I activation [51]. Furthermore, mTORC1 indirectly regulates Pol III to promote expression of tRNA; mTORC1 does this by phosphorylation and inactivation of Maf1, a negative repressor of Pol III [52]. mTORC1 also associates with the promoters of Pol I and Pol III to directly drive their transcription [53]. So, via multiple mechanisms, mTORC1 enhances ribosomal biogenesis to enhance the efficiency of protein translation and cell growth, which often becomes dysregulated in cancer.

### 2.3. Metabolic Transformation by mTORC1

**Figure 2:**
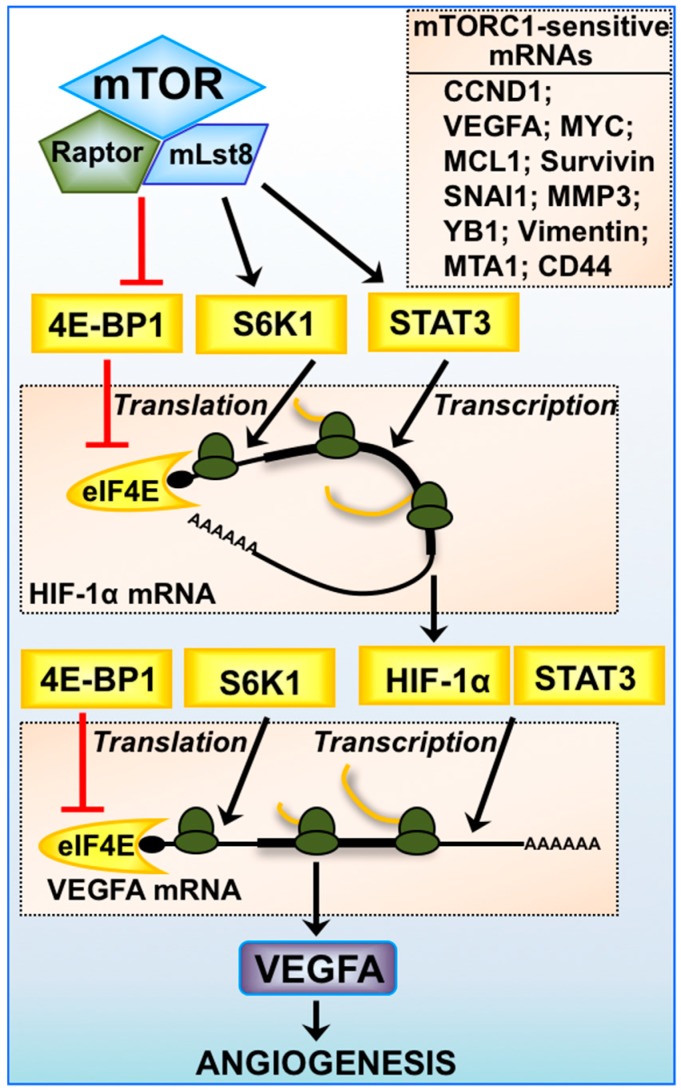
Expression of mTORC1-sensitive mRNAs. mTORC1-mediated regulation of angiogenesis via HIF-1α and vascular endothelial growth factor A (VEGFA) is multifaceted, where transcription of HIF-1α mRNA is driven by STAT3 and VEGFA mRNA by both HIF-1α and STAT3. Protein translation of HIF-1α and VEGFA mRNA is highly dependent on the availability of eIF4E and the activity of S6K1. The 5′-untranslated region (UTR) of VEGFA mRNA is highly structured and is considered to be a 5′-TOP mRNA. Other mTORC1-sensitive mRNAs involved in cancer progression are listed: cyclin D1 (CCND1), myelocytomatosis (MYC), myeloid cell leukemia sequence 1 (MCL1), Survivin, snail family zinc finger 1 (SNAI1), matrix metalloproteinase-3 (MMP3), Y-box binding protein 1 (YB1), Vimentin, metastasis associated 1 (MTA1), and clusters of differentiation 44 (CD44).

By altering their metabolism to favour aerobic glycolysis, cancer cells fulfil their bioenergetics and biosynthetic demands to elicit a proliferative advantage (reviewed in [54]). This phenomenon was first described by Otto Warburg in 1924, who discovered that proliferative cancer cells consumed glucose at an elevated rate and released lactic acid rather than CO_2_ [55,56]. This finding revealed that some cancer cells favoured aerobic glycolysis over mitochondrial oxidative phosphorylation in conditions when oxygen is not limited, a term coined the “Warburg effect”. Given that aerobic glycolysis generates a lot less adenosine triphosphate (ATP) per glucose molecule when compared to oxidative phosphorylation (only 5% of glucose's energy potential: producing two ATP molecules rather than 38), such a dramatic adjustment to how glucose is metabolised might first appear counterintuitive to a cancer cell. However, to generate more energy during aerobic glycolysis, a cancer cell can increase the rates of glucose uptake to meet its energy demand. The “Warburg effect” provides a proliferative advantage to a cancer cell when the generation of energy is not rate-limiting. Metabolic rewiring enhances entry of glucose into the pentose phosphate pathway to generate nicotinamide adenine dinucleotide phosphate, ribose-5-phosphate, and erythrose-4-phosphate, which are precursors for fatty acids, nucleotides and aromatic amino acids, respectively. Such precursors are essential for a hyper-proliferative cancer cell, allowing rapid anabolic growth by *de novo* synthesis of membranes, rRNA, mRNA, DNA, and proteins (see Figure 2, and reviewed in [3]).

While there are several ways that a cancer cell can acquire pyrimidine and purine nucleotides, the most efficient way is through the pentose phosphate pathway. Nucleotide precursors are not only essential for DNA replication, but they are also needed for the generation of mRNA (to make proteins) and rRNA (for rRNA processing and the production of ribosomal proteins). Within the pentose phosphate pathway, mTORC1 further enriches the pool of pyrimidine nucleotides through S6K1. S6K1 phosphorylates carbamoyl-phosphate synthetase 2, aspartate transcarbamylase, and dihydroorotase (CAD), a trifunctional enzyme that catalyses the first three enzymatic steps (of a 6-step process) within the pyrimidine biosynthesis pathway [7,57]. Furthermore, mTORC1 was recently found to upregulate the biosynthesis of purines via a transcriptional mechanism, where methylenetetrahydrofolate dehydrogenase 2 (MTHFD2) expression was enhanced by mTORC1 [58]. mTORC1 was found to promote ATF4 protein synthesis, leading to enhanced gene-expression of MTHFD2, a metabolic enzyme involved in the promotion of purine synthesis [58]. ATF4 is a member of the CREB/ATF family of bZIP transcription factors and is classically known to be upregulated in response to nutrient starvation, ER stress and mitochondrial dysfunction. ATF4 enhances cell survival during periods of cell stress. However, ATF4 is also necessary for homeostatic balance of a growing cell to help maintain the supply of amino acids. As well as the regulation of metabolic enzymes, ATF4 regulates the gene-expression of amino acid transporters and tRNA aminoacyl transferases involved in charging tRNAs with their cognate amino acids. mTORC1’s involvement in the regulation of ATF4 translation as a mechanism to enhance cell growth makes perfect sense. Through ATF4, mTORC1 effectively regulates the biosynthesis of purines as well as the uptake and delivery of amino acids to the translation machinery [59]. In the cancer setting, mTORC1/ATF4 is likely to contribute to metabolic transformation. It should be noted that higher levels of MTHFD2 expression is often observed in many cancers and correlates to poor survival in breast cancer [60].

Another critical way that mTORC1 promotes metabolic adaption is through the activation of the oxygen-sensitive transcription factors, Hypoxia Inducible Factor-1 α (HIF-1α) and HIF-2α (Figure 2). Through HIF-dependent gene-expression, a cell’s metabolic state can switch from oxidative phosphorylation to glycolysis. Typically, the role of HIF is to increase the ability of a cell to survive during conditions when oxygen becomes limited. Cancer cells take advantage of many features associated with HIF activation. As well as promoting cell survival, HIF promotes glucose uptake, angiogenesis, proliferation and metastasis. High levels of HIF protein expression often correlates with an increased risk of mortality in many cancer types (reviewed in [61]). The stability of the α-subunit of HIF is regulated by oxygen. When oxygen levels are high, two proline residues within the oxygen-dependent degradation domain of HIF-1α are hydroxylated by oxygen-dependent prolyl hydroxylase domain proteins. Proline hydroxylation results in ubiquitin-mediated degradation of HIF-α and requires the tumour suppressor protein, Von Hippel-Lindau (reviewed in [62]). Consequently, in conditions of low oxygen tension, HIF-1α protein is stabilised that then functions as a heterodimer with HIF-1β to drive gene-expression of target genes with hypoxia response elements. The regulation of HIF-1α by mTORC1 is multifaceted. While the stability of HIF-1α is not regulated by mTORC1, its protein translation is acutely regulated by both the availability of eIF4F and the activity of S6K1, placing mTORC1 as central driver of HIF [63]. Furthermore, mTORC1 indirectly enhances the transcription of HIF-1α mRNA via STAT3. Research using Tuberous Sclerosis disease models has shown a tight correlation between mTORC1 and HIF activation. Loss of TSC2 was found to induce a 7-fold increase in HIF-1α transcriptional activity in conditions of hypoxia, which was partially restored upon treatment with rapamycin [63]. In another study, gene-expression arrays showed an elevation in expression of HIF-regulated metabolic genes in *Tsc2*−/− mouse embryonic fibroblasts (MEFs) that was rescued with rapamycin treatment [64]. Such work highlights the impact that loss of TSC2 and mTORC1 activation can have on HIF.

STAT3 signalling is necessary to promote angiogenesis through HIF. It was shown that STAT3 knockdown completely ablate expression of HIF-1α, HIF-2α and VEGFA [63]. Furthermore, STAT3 is a downstream target of mTORC1 (Figure 2) [63]. STAT3 is a member of the STAT protein family, a group of latent transcription factors (STAT-1, 2, 3, 4, 5a, 5b and 6) that become activated in response to either cytokine or growth factor interactions with cell membrane receptors (reviewed in [65]). STAT3 is activated by ligand binding to the interleukin 6 (IL-6) receptor family members, causing recruitment and activation of Janus kinase (JAK) family members [66]. STAT3 contains two characterised phosphorylation sites, Tyr705 and Ser727 that are both required to be phosphorylated for its full activation. JAK phosphorylates STAT3 at Thr705, which is required for its translocation to the nucleus to upregulate cytokine mediated gene expression [67]. mTORC1 has been shown to directly phosphorylate STAT3 at Ser727 [63] and to be partially sensitive to rapamycin treatment. STAT3 is classed as an oncogene and plays a pivotal role in carcinogenesis and tumour formation. Several STAT3 target genes are reported to be upregulated during tumour formation including B-cell lymphoma-extra large (Bcl-XL), Survivin, Hsp70, CCND1, MYC, HIF, and VEGFA [68], where STAT3 orchestrates the angiogenic response through HIF and VEGFA. Many signalling pathways converge on STAT3, including mTORC1, and in cancer they are known to drive malignancy. 

### 2.4. mTORC2 Signalling and Cancer

Historically, the functional differences between mTORC1 and mTORC2 have been difficult to tease apart because of the conservation in critical mTOR complex components, and signalling cross-talk between the two complexes. Early studies involving genetic or pharmacological inhibition, particularly with rapamycin, led to conflicting results and confusion. It is now clear that these discrepancies occurred because of indirect effects of long-term treatment with rapamycin that leads to the sequential inhibition of mTORC2 assembly [17]. Although mTORC1 was first characterised as an upstream regulator of serum/glucocorticoid regulated kinase 1 (SGK1) [69], in fact mTORC2 is the *bona fide* hydrophobic motif kinase controlling SGK1 activation by Ser422 phosphorylation [70]. This finding was confirmed with the use of different mTOR drug inhibitors, where Ku-0063794 (an ATP-competitive inhibitor that blocks both mTORC1 and mTORC2) could inhibit SGK1 activation, while rapamycin was not [70,71].

Mechanistically, SGK1 interacts with SIN1 and likely also PROTOR-1 in the mTORC2 complex, and both proteins are required for phosphorylation of Ser422 and activation of SGK1, leading to phosphorylation of downstream substrates such as N-myc downstream regulated 1 (NDRG1) and epithelial sodium channel (ENaC) [72,73]. Phosphorylation of the mTORC2-specific component, Rictor at Thr1135 in response to amino acids and growth factors occurs through mTORC1-dependent activation of ribosomal protein S6K1 [74]. Thr1135 phosphorylation does not lead to major changes in mTORC2-kinase activity, but may be important for switching mTORC2 substrate specificity. In *Rictor*−/− MEFs, SGK1 expression is increased, whereas in wild-type cells mTORC2-dependent phosphorylation of SGK1 at Ser422 leads to activation but then subsequent turnover of the kinase [75]. Rictor inhibition in pancreatic cancer leads to impaired tumour growth and phosphorylation of AGC kinases, including SGK1 [76]. DEPTOR is an mTOR-binding protein that inhibits mTORC2 signalling, so it is not surprising to find that DEPTOR expression is dramatically reduced in many tumour tissues, including oesophageal squamous cell cancer [77]. Ectopic DEPTOR expression suppresses cellular proliferation, migration, and invasion phenotypes, concomitant with reduced phosphorylation of SGK1 and NDRG1 [77]. However, DEPTOR overexpression occurs in a subset of multiple myelomas with cyclin D1/D3 or c-MAF/MAFB translocations. In this context, DEPTOR suppresses S6K1 but, by relieving feedback inhibition from mTORC1 to PI3K signalling, activates AKT signalling [13]. Thus, the regulation of AGC kinase activation by mTORC2 complexes is highly convoluted, and is dictated by both gene-expression and cell context.

mTORC2, SGK1 and oncogenesis: therapeutic resistance is one of the major obstacles in the effective treatment of cancer patients, of which alkylating chemotherapy is often the standard of care. In glioma, resistance to drugs such as temozolomide are mediated through the DNA repair protein O6-methylguanine-DNA methyltransferase (MGMT) [78]. Activation of mTORC2 and thus SGK1-phosphorylated NDRG1 is increased in temozolomide resistant glioma cell lines and NDRG1 expression is elevated in tissues specimens from glioma patients, suggesting that this is mechanistically important for MGMT-conferred resistance to alkylating therapeutics [78].

Resistance to PI3K pathway inhibition is an emerging barrier to effective use of molecularly targeted therapies and may in part be explained by the reported increases in SGK1 expression and activity. For example, AKT-inhibitor (AZD5363 and MK-22060) resistant cancer cell lines show increased NDRG1 phosphorylation, demonstrating that SGK1 can compensate for PI3K pathway inhibition [79]. In colorectal cancer stem cells obtained from patients, mTORC2 expression is elevated, compared to mTORC1 and this correlates with enhanced SGK1 activity [80]. Knockdown of SGK1 in those cells decreased growth, invasiveness, and chemoresistant properties. The Heterogeneous nuclear ribonucleoprotein M (HNRNPM) binds to Rictor in the mTORC2 complex to enhance activation of AGC kinases, including SGK1, at least in muscle [81], but this protein is also involved in cancer invasion and metastasis [82,83]. So, while it remains to be confirmed, enhanced activation of SGK1 through increased expression of HNRNPM may contribute to oncogenic phenotypes. SGK1, acting downstream of mTORC2, may also function as a cell survival kinase by regulating the stability of the TP53 E3 ubiquitin ligase human double minute 2 (HDM2) protein [84].

The androgen receptor (AR) plays a pivotal role in prostate cancer growth and androgen is known to exert its effects, in part by stimulating mTORC2 activation [85]. Conzen et al. highlighted the importance of cancer cell context because they reported that rapamycin-mediated growth inhibition and inactivation of insulin-mediated SGK1 phosphorylation depends on Estrogen receptor alpha (ERα) status in breast cancer cells [86]. This highlights the complexity of SGK1 regulation, which is likely cell-type specific and dependent on multiple cross-talk mechanisms, especially when drawing conclusions from the use of rapamycin, since it also interferes with mTORC2 complexes [17]. Although the weight of evidence supports the importance of SGK1 in cancer, exceptions do occur. For example, in multi-drug resistant tongue cancer, miR-491-3p, which regulates Rictor expression is downregulated, leading to decreased mTORC2 activity and phosphorylation of the hydrophobic motif of SGK1 [87]. However, profiling of cancers for developing PI3K pathway resistance is likely to be an effective way of identifying which particular patient cohorts will be predisposed to SGK1-mediated resistance that could be treated with additional drugs that target mTORC2/SGK1.

Finally, in cancer immunotherapy approaches, T-cell activation and enhancement of T helper type 1 (TH1) cell-mediated immune functions play a crucial part of a robust in anti-tumour response. SGK1 is known to promote TH2 differentiation by preventing the degradation of the transcription factor JunB, which is mediated by the E3 ligase Nedd4-2 [88]. In addition, SGK1 activity regulates the transcription factor TCF-1 to repress interferon-γ (IFN-γ) production. In mice, T-cell specific deletion of SGK1 results in the animals being more capable of rejecting tumours [88]. Therefore, anti-cancer therapies that target SGK1 may have the additional beneficial effect of increasing pools of Th1 cells to enhance adaptive immunity-mediated anti-tumour responses.

### 2.5. mTOR Inhibitors to Treat Cancer

Rapamycin was isolated from the bacteria *Streptomyces hygroscopius* in the early 1970’s, discovered in Easter Island (or as the natives call it, “Rapa Nui”) (for review see [89]). Rapamycin (drug later named as Sirolimus) was originally defined as an antifungal compound, but was subsequently found to be much more effective as an immunosuppressant with anti-proliferative properties. Due to these anti-proliferative properties, cancer researchers have had much interest in the drug target of rapamycin, mTOR. As examples of mTOR’s involvement in a non-cancerous setting, mTOR-driven proliferation of keratinocytes helps facilitate wound healing [90] and is also necessary as a key metabolic regulator to drive an immune response (reviewed in [91]). Rapamycin exerts immunosuppressive effects by limiting the proliferation of T-lymphocytes and is currently approved for treatment of transplant patients to prevent graft rejection. Therefore, side-effects when using rapamycin-based drugs that inhibit mTORC1 can often compromise or delay wound healing, cause immunosuppression and consequently increase the risk of infection.

Much of our basic understanding of mTOR is based on research using rapamycin, which has functioned as an essential research tool for delineating the complexities of mTOR signalling as well as cell processes regulated by mTOR. Rapamycin binds to an immunophilin, FKBP12 (12 kDa FK506 binding protein), and as a drug-protein complex allosterically inhibits mTORC1 by binding to the FKBP12-rapamycin binding (FRB) domain that is opposite to the catalytic domain of mTORC1. It should be noted that rapamycin is an incomplete inhibitor of mTORC1, as some mTORC1-dependent processes are rapamycin-insensitive [92]. As an example, the first two priming phosphorylation sites of 4E-BP1 that are mediated by mTORC1 (Thr37/Thr46) are heavily resistant to rapamycin treatment [93]. Autophagy is a catabolic process that directly opposes anabolic cell growth. mTORC1 modulates autophagy through the phosphorylation and destabilisation of unc-51-like autophagy activating kinase 1 (ULK1) [94], the kinase responsible for autophagy induction. There is conflicting evidence that mTORC1-dependent suppression of autophagy is completely sensitive to rapamycin (see [4] for a detailed review), although compounds that inhibit mTORC1 and mTORC2 confirm the importance of mTOR signaling in autophagy regulation [92,94].

The poor solubility and pharmacokinetics of rapamycin (Sirolimus) triggered the development of several rapamycin analogues (rapalogues) [95] and see Table 1. Two water-soluble rapalogues, temsirolimus (developed by Wyeth-Ayerst/Pfizer) and everolimus (developed by Novartis), were approved by the Food and Drug Administration in 2007 and 2009 for the treatment of advanced renal cell carcinoma (RCC) [96] and mantle cell lymphoma [97], respectively. Everolimus is now also being used to treat neuroendocrine tumours, gastric cancer, TSC- and neurofibromin 1 (NF1)-related tumours (reviewed in [98]). Growth of tumours in RCC is highly dependent on mTORC1, HIF, and VEGF, that drive a pro-angiogenic response. In the microenvironment of the kidney, angiogenic signalling is crucial for metabolic transformation and malignancy. The critical involvement of mTORC1 in RCC is evident with the current allosteric inhibitors of mTORC1, temsirolimius and everolimus. With temsirolimius, the median overall survival of patients with RCC was 10.9 months [99]. While with everolimus, survival was observed to be increased by 5.9 months in advanced RCC patients who previously failed treatment with either of the anti-angiogenic agents, sorafenib or sunitinib [100].

There is much clinical interest, with more than 400 registered trials (clinicaltrials.gov) using rapalogues as well as second generation inhibitors (the ATP-competitive inhibitor category of mTOR inhibitors) to treat many cancer types, such as breast, melanoma, myeloma, renal, gynecological, and brain cancers, as a mono agent or in combination. As an example of a combinatory trial, there is a phase 1b/2 clinical trial using chemotherapy in the presence with both Everolimus with Lapatinib (a dual tyrosine kinase inhibitor that inhibits both HER2 and EGF receptors) to treat metastatic HER-2 positive breast cancer (ClinicalTrials.gov Identifier: NCT01783756). These second-generation mTOR inhibitors acts as ATP competitors, binding within the ATP-binding pocket of mTOR, preventing the activity of both mTORC1 and mTORC2 (Table 1). This class includes MLN0128, (TAK-228), an ATP-competitive inhibitor of mTOR that is currently being tested in 37 clinical trials at both phase 1 and 2 [103]. This class of mTOR inhibitor is much more effective at blocking mTORC1 activity when compared to rapamycin. Given the wide network of mTORC1 targets that are involved in cancer, it is surprising that mTOR inhibitors are not more widely used for treating cancer. The reason behind mTOR inhibitors having limited clinical success is that their mechanism of action is cytostatic rather than cytotoxic, which can lead to acquired resistance. mTORC1 inhibition can lead to cell survival through induction of autophagy and can limit the effectiveness of the therapy. Another reported mechanism of drug resistance is through mutation, either within the FRB domain or the kinase domain of mTOR [101]. To solve this issue of drug resistance, a third generation mTORC1 inhibitor (RapaLink-1) was developed, which can simultaneously associate with and allosterically inhibit mTORC1 via the FRB domain while also binding within the ATP-binding pocket of mTOR to block the catalytic activity of mTORC1 [107]. This third generation mTORC1 inhibitor was developed after Rodrik-Outmezguine and her group. By chronically exposing two breast cancer cell lines (MCF-7 and MDA-MB-468) to rapamycin [107], they observed that these cells acquired resistance to rapamycin. Resistance was caused by mutations within the FRB domain and the kinase domain of mTOR, resulting in mTOR hyper-activation. It was observed that the drug association of rapamycin and AZD8055 (ATP-competitive inhibitor) to mTOR were proximal to one another. This unique juxtaposition of the two drugs led to the idea and then the development of RapaLink-1. When compared to rapamycin and the second generation mTOR inhibitors, RapaLink-1 shows a higher efficiency to target and inhibit mTORC1. Consequently, RapaLink-1 has better efficacy to inhibit proliferation in both cell and xenograft models. Even though RapaLink-1 is still at the pre-clinical stage, RapaLink-1 holds much promise in the treatment of mTOR-hyperactive cancers.

## 3. Conclusions

mTOR is the master regulator of cell growth control, where oncogenic mTOR signalling through both complexes commonly occur in cancer. In part, mTORC1 drives cell growth at the level of protein translation. Enhanced translation of mTORC1-sensitive mRNA transcripts play a critical role in promoting cell growth and has the capability to transform cells. mTORC1 is not just limited to the regulation of translation factors, but also regulates transcriptional events involved in ribosomal biogenesis, metabolic transformation and cell cycle progression. While less is understood regarding mTORC2, its involvement in cancer progression is beginning to emerge. With mTOR having a prominent role in cancer progression, it is initially surprising that mTOR inhibitors have had less clinical impact to treat cancer. This is due to the cytostatic nature of mTOR inhibitors. RapaLink-1 has much promise in the treatment of cancer as well as future combination therapies. Another possible solution is not to target mTOR at all, but instead to exploit vulnerabilities within those cancers that have an mTOR-driven oncogenic signature. There is clearly much we still need to know regarding how mTOR modulates the control of cell growth. Given what we know to date, it is probable that both mTOR complexes will influence most signalling processes that are linked to cell growth control.

## Figures and Tables

**Table 1:** mammalian/mechanistic Target of Rapamycin (mTOR) inhibitors.

First Generation Inhibitors		References
Rapamycin/sirolimus	The first, and most widely reported mTOR inhibitor. Rapamycin interacts with FKBP12 to interfere with mTOR substrate recognition, with IC50 values reported < 1 nM.	[89,92]
Temsirolimus (CCI-779)	A rapalog generated by replacing the hydrogen at C-40-O position with dihydroxylmethyl propionic acid ester. Inhibition is mechanistically similar to rapamycin/sirolimus with IC50 values of < 1 nM.	[95,96,101]
Everolimus (RAD001)	This rapalog has a hydroxylethyl group replacing the C-40-O hydrogen and is also mechanistically similar to rapamycin/sirolimus with IC50 values of < 1 nM.	[95,97,102]
**Second Generation Inhibitors**		
MLN0128 (TAK-228)	Potent and selective ATP-competitive of mTOR kinase with in vitro IC50 of 1 nM.	[103]
AZD8055	Potent and highly selective ATP-competitive inhibitor of the mTOR kinase subunit with an IC50 of approximately 0.8 nM in cells	[104]
KU-0063794	Potent and highly selective inhibitor of the mTOR kinase subunit with an IC50 of approximately 10 nM.	[105]
**Third Generation Inhibitors**		
Rapalink-1	Hybrid of first and second generation mTOR inhibitors that takes advantage of the two original docking sites thus creating a bivalent interaction that circumvents resistance developed against the original compounds.	[106]
